# SegFormer-based nectar source segmentation in remote sensing imagery

**DOI:** 10.3389/fpls.2025.1666619

**Published:** 2025-10-01

**Authors:** Mengting Dong, Hao Cao, Tian Zhao, Xu Zhao

**Affiliations:** College of Information and Network Engineering, Anhui Science and Technology University, Bengbu, Anhui, China

**Keywords:** remote sensing, SegFormer, nectar-producing plants, bees, semantic segmentation, deep learning

## Abstract

**Introduction:**

Beekeepers often face challenges in accurately determining the spatial distribution of nectar-producing plants, which is crucial for informed decision-making and efficient beekeeping.

**Methods:**

In this study, we present an efficient approach for automatically identifying nectar-producing plants using remote sensing imagery. High-resolution satellite images were collected and preprocessed, and an improved segmentation model based on the SegFormer architecture was developed. The model integrates the CBAM attention mechanism, deep residual structures, and a spatial feature enhancement module to improve segmentation accuracy.

**Results:**

Experimental results on rapeseed flower images from Wuyuan County demonstrate that the improved model outperforms the baseline SegFormer model. The mean Intersection over Union (mIoU) increased from 89.31% to 91.05%, mean Pixel Accuracy (mPA) improved from 94.15% to 95.02%, and both mean Precision and mean Recall reached 95.40% and 95.02%, respectively.

**Discussion:**

The proposed method significantly enhances the efficiency and accuracy of nectar plant identification, providing real-time and reliable technical support for precision beekeeping management, smart agriculture, and ecological monitoring. It plays a key role in optimizing bee colony migration, improving collection efficiency, and regulating honey quality.

## 1 Introduction

**Figure 1:**
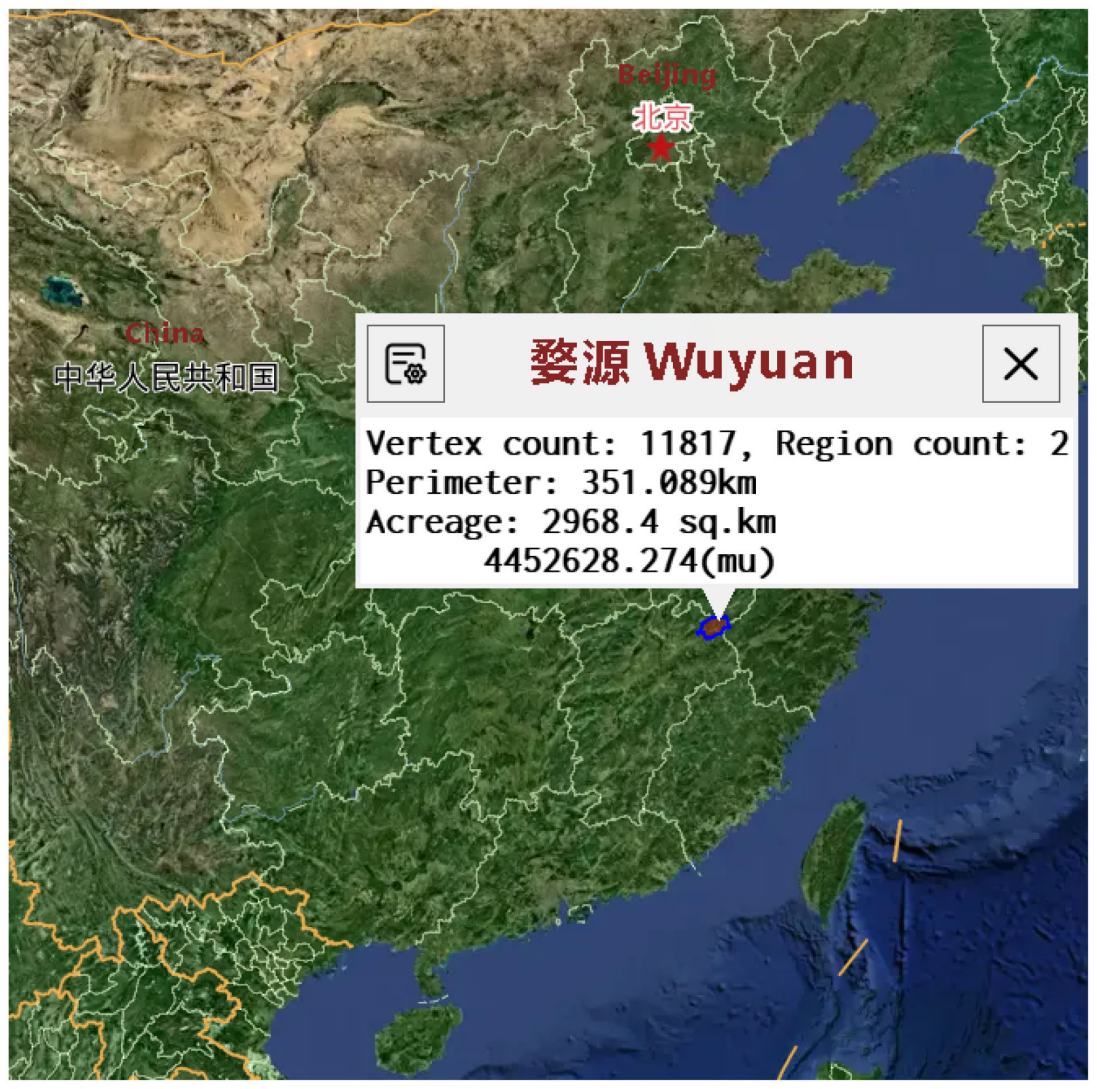
Basic information of the dataset selection area.

Nectar-producing plants serve as the foundational ecological resource for the beekeeping industry, directly influencing honey yield and quality through their species composition, distribution density, and phenological characteristics (Khan and Khan, 2018). Precisely analyzing the spatial distribution patterns of nectar-producing vegetation is crucial for optimizing bee colony migration routes, improving honey collection efficiency, and regulating honey’s nutritional quality (Ma and Yang, 2024). The sustainable development and utilization of nectar-producing resources are critical issues for the high-quality advancement of the beekeeping industry (Hunde, 2025). This study uses Wuyuan County, Jiangxi Province, as a representative area for nectar-producing plant research. Its favorable ecological conditions, including a warm, humid climate and abundant water resources, support the growth of various nectarproducing plants, particularly rapeseed flowers, which serve as a primary nectar source for bees (Abrol et al., 2007). The extensive cultivation of rapeseed flowers has not only contributed significantly to the 3 local agricultural economy but has also provided a vital resource for the beekeeping industry, attracting numerous tourists and fostering the diversified growth of the regional economy (Jiang et al., 2016). As shown in **Figure 1**, the specific location of Wuyuan County is an ideal site for nectar source research, particularly given its unique geographical features and abundant nectar-producing plant resources.

However, the complex terrain of Wuyuan and its seasonal variations, which include fluctuations in climate, temperature, and precipitation, pose significant challenges for the remote sensing identification of nectar-producing plants, as these changes directly affect the growth cycles of the plants (Barahona et al., 2024). Traditional methods for identifying nectar-producing plants primarily rely on human expertise and typically require field surveys to ascertain the location and extent of nectar sources (Langlois et al., 2020). While effective on a small scale, this method becomes time-consuming and labor-intensive when applied to large areas or mobile beekeepers. It is also vulnerable to environmental changes, hindering real-time monitoring of the growth and distribution of nectar-producing plants (Zheng et al., 2018). These methods depend on predictable environmental patterns, such as temperature and rainfall, which are increasingly disrupted by climate change. These methods rely on predictable environmental patterns, such as temperature and rainfall, which are becoming increasingly erratic due to climate change (Vercelli et al., 2021). Furthermore, traditional methods are ill-equipped to handle seasonal variations and are unable to quickly adjust recognition models to accommodate dynamic environmental changes, particularly when beekeeping sites migrate seasonally, making fixed monitoring methods even more limited. In contrast, remote sensing technology, particularly when combined with deep learning algorithms, has been widely applied in agriculture (Khanal et al., 2020). Especially when combined with deep learning algorithms, remote sensing technology can rapidly and accurately extract and identify the spatial distribution information of nectar-producing plants over a large area, making it of significant importance in the large-scale monitoring of nectar-producing plant distributions (Barnsley et al., 2022). Through drone or satellite remote sensing imagery, remote sensing technology can efficiently and precisely extract spatial distribution information from nectar-producing plants, addressing the limitations of traditional methods (Namdeo Aiwale et al., 2025). Nevertheless, complex terrain backgrounds and variations in the size of nectar-producing plants make remote sensing image processing more challenging, especially when environmental conditions are complex and plant growth states vary significantly. Existing remote sensing identification methods often face low accuracy and segmentation precision under such conditions.

To address issues such as multifaceted backgrounds and varying target scales encountered during the segmentation of nectar-producing plants in remote sensing images, this paper proposes a segmentation method based on an improved SegFormer model. This method fully leverages the SegFormer model’s advantages in multi-scale feature extraction and self-attention mechanisms, enabling efficient processing of diverse plant distribution scenarios and making it suitable for the automatic identification of nectarproducing plants. To further enhance the segmentation accuracy of the model, this paper introduces the Convolutional Block Attention Module mechanism and Spatial Attention mechanism into the SegFormer architecture, combined with deep residual technology. CBAM effectively uncovers correlations between different channels, enhancing the model’s ability to represent plant regions (Zhang et al., 2023); the Spatial Attention mechanism helps the model focus on key areas in the image, thereby improving segmentation accuracy (Zhu et al., 2019). Additionally, through the deep residual structure, the model effectively enhances feature expression capabilities and gradient propagation, improving segmentation performance for nectar-producing plants of various sizes and shapes (Fang et al., 2021). Whether in densely populated rapeseed flower areas or in dispersed vegetation regions, the model achieves high-precision segmentation.

The application of remote sensing image segmentation technology in the large-scale monitoring of nectar plant distribution is of great significance (Adgaba et al., 2017). With the development of the beekeeping industry, mobile beekeeping has gradually become a common farming method. Beekeepers need to quickly and accurately understand the distribution of nectar-producing plants in different regions to make scientific honey harvesting decisions and flexibly adjust beekeeping strategies (Harianja et al., 2023). This helps beekeepers adjust their honey harvesting plans in real time, thereby improving honey production and quality. With technological advancements, the application of remote sensing technology and deep learning methods in nectar-producing plant monitoring has become increasingly precise and efficient (Hicks et al., 2021). This enables beekeepers not only to obtain real-time spatial distribution information but also to conduct rapid monitoring across large areas, achieving more flexible and scientific honey harvesting decisions. Through this technology, beekeepers can promptly understand the distribution of nectar-producing plants in different regions, adjust beekeeping strategies and honey harvesting plans, and maximize honey production and quality. This not only helps the beekeeping industry improve production efficiency but also provides strong technical support and theoretical foundations for the development of precision agriculture.The remainder of this paper is structured as follows. Section 1 introduces the experimental materials, including the methods for data acquisition, preprocessing, and dataset construction. Section 2 elaborates on the remote sensing image honey source segmentation model based on the improved SegFormer, detailing the fundamental SegFormer architecture and the introduced enhancements: the CBAM attention mechanism, deep residual structure, and spatial feature enhancement module. The novelty of this work lies in the integration of these well-established techniques to address the unique challenges in nectar plant segmentation in remote sensing imagery. Although CBAM, spatial attention, and residual blocks are widely known in the computer vision domain, their combination and application to nectar plant segmentation is a key contribution of this study. This integrated approach enables more effective segmentation in intricate environments, enhancing model robustness and accuracy. Section 3 describes the model training environment, evaluation metrics, and validates the effectiveness of the improved model through ablation studies and comparative experiments. Finally, Section 4 summarizes the main conclusions of this study and discusses potential future research directions.

## 2 Experimental materials

### 2.1 Image acquisition and processing

In this study, 621 remote sensing images were obtained from the Jilin-1 satellite via the Aokang Interactive Map Platform. The images cover Wuyuan County in northeastern Jiangxi Province, with acquisition dates from March 2022 to May 2025. The satellite imagery includes various resolutions (500 meters, 100 meters, and 50 meters), with the 50-meter resolution images serving as the primary data source. The 50-meter resolution images, which represent the highest precision obtained from the platform under current conditions, strike a balance between spatial resolution and coverage, making them particularly suitable for large-scale environmental monitoring and agricultural research. This resolution allows for monitoring vast areas, such as agricultural land or natural ecosystems, providing sufficient detail to study plant distribution, vegetation health, and land use changes. The Jilin-1 satellite can acquire various types of remote sensing images, including optical and multispectral images, providing valuable data support for the identification of nectar-producing plants (Li et al., 2021). To ensure the images meet the input requirements of deep learning models, all images underwent a series of preprocessing and enhancement steps.

During image preprocessing, the original remote sensing images were first randomly cropped to extract different regions of interest, with the crop size ranging from 80% to 100% of the original image. This random cropping helps increase the diversity of the dataset and allows the model to learn from various parts of the image, improving its robustness. The cropped images were then normalized, and their size and aspect ratio were standardized to meet the input requirements of the deep learning model. The image size was adjusted to 512×512 pixels to reduce computational resource consumption during training while ensuring that important image details were preserved. Subsequently, various random image enhancement techniques were applied to further augment the dataset, including brightness and contrast adjustments (random values between -20% and +20%), random rotation (between -30° and +30°), random translation (with a maximum offset of 10% of the image size), mirror flipping, and noise addition (with a variance of 0.01).

Through the above processing and enhancement steps, the original 621 remote sensing images underwent seven rounds of enhancement processing, resulting in a total of 4,337 images used for model training. These enhanced images not only expanded the scale of the dataset but also improved the model’s generalization ability, enabling it to better handle the task of identifying nectar-producing plants under complex environmental conditions and seasonal changes.

### 2.2 Dataset creation

**Figure 2:**
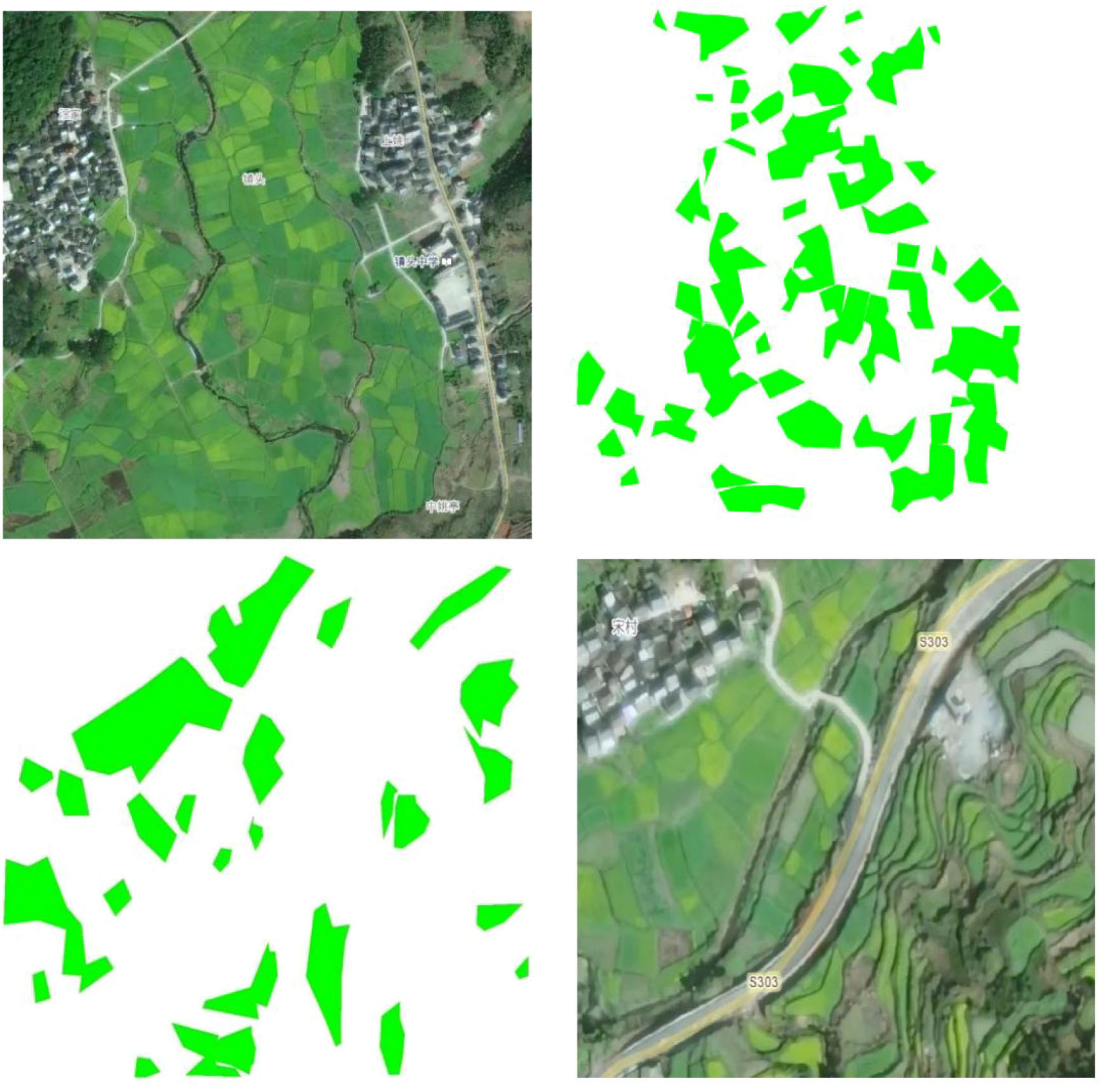
Nectar plant segmentation: original image vs. segmentation mask.

The images were annotated using Labelme annotation software. Based on the typical color characteristics of rapeseed flowers in remote sensing images, the areas containing rapeseed flowers were initially preselected using color values in the range of RGB (200–255, 200–255, 0–100). All nectarproducing plant regions were precisely labeled, and corresponding annotation files in JSON format were generated. After annotation, the dataset was randomly divided into training, validation, and test sets with a ratio of 8:1:1. This ratio helps balance the adequacy of training data with the effectiveness of model evaluation. Subsequently, all annotated JSON files were converted into mask images for use in training deep learning models. Each mask image was a binary image, with the same size as the original image, where annotated areas were assigned a value of 1 and the background was assigned a value of 0. This conversion process was automated through a script to ensure accurate generation of the corresponding mask images from the annotations, providing high-quality labeled data for subsequent image segmentation tasks. The images and their corresponding masks are shown in **Figure 2**.

## 3 Methods

### 3.1 SegFormer model

SegFormer is an efficient image segmentation model based on the Transformer architecture, particularly suited for handling semantic segmentation in complex backgrounds and large-scale regions (Xie et al., 2021). Unlike traditional convolutional neural networks (CNNs), SegFormer leverages selfattention mechanisms and multi-scale feature extraction to effectively capture long-range dependencies in images, making it highly suitable for scenarios in remote sensing images where target sizes and scales vary significantly (Huang et al., 2023). The model employs a novel hierarchical Transformer encoder that outputs multi-scale features, extracting information from different levels through progressive downsampling to fully preserve spatial and contextual features (Wang et al., 2022). Additionally, SegFormer does not use positional encoding, avoiding performance degradation caused by interpolation when resolution changes (Huang et al., 2021). The model also simplifies the decoding stage by using a lightweight MLP decoder, which effectively aggregates features from different layers to fuse local and global information (Shi et al., 2022). This design improves model inference efficiency while maintaining segmentation accuracy.

**Figure 3:**
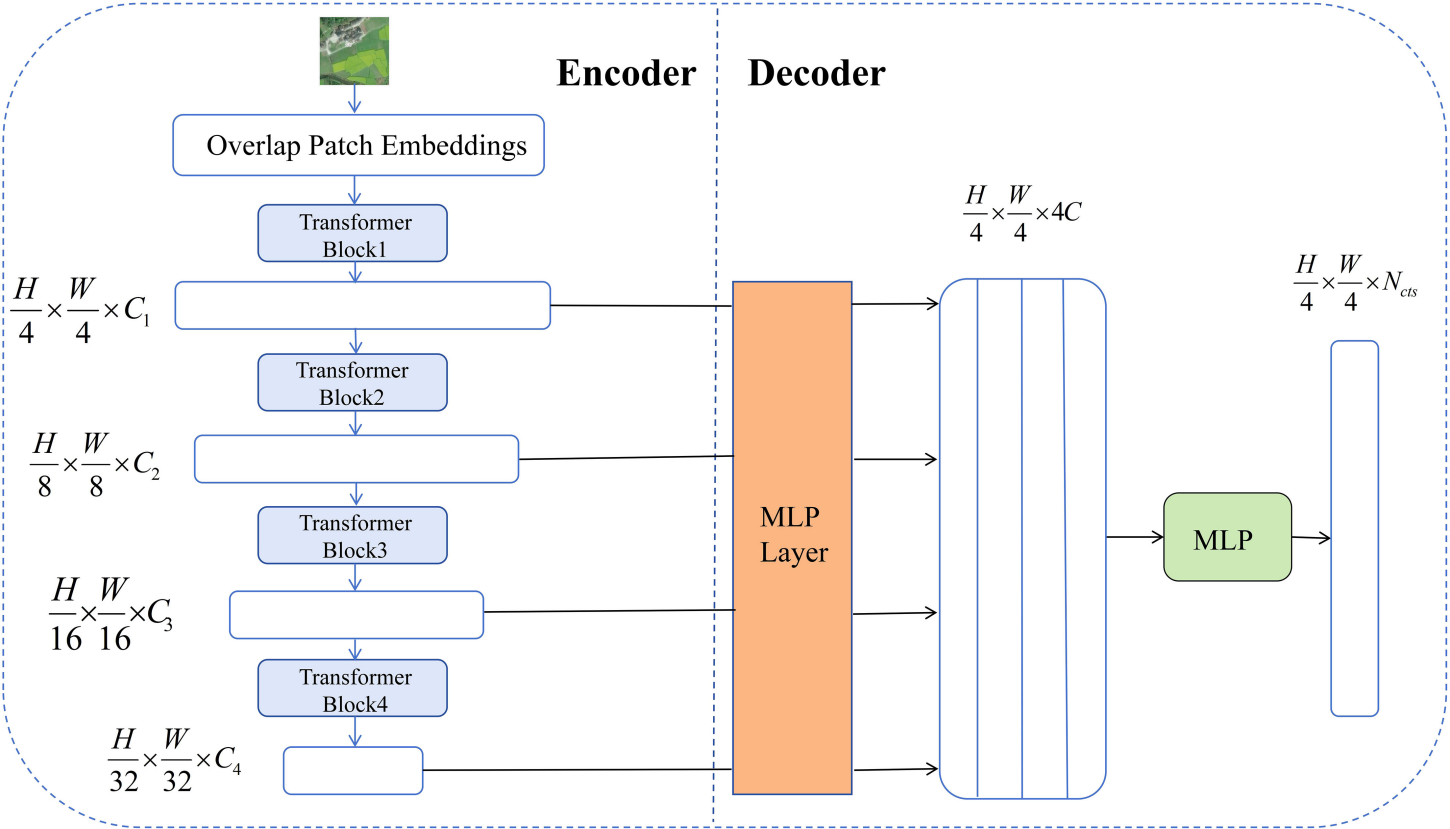
SegFormer architecture with enhanced channel attention for nectar plant segmentation.

Overall, SegFormer demonstrates exceptional multi-scale feature fusion and representation capabilities, effectively addressing challenges such as complicated terrain features, diverse targets, and inconsistent scales in remote sensing image segmentation tasks. Leveraging the powerful modeling capabilities and efficient architecture of the Transformer framework.In remote sensing image segmentation tasks, it is often necessary to distinguish between different types of land cover (such as vegetation, water bodies, buildings, etc.), with objects exhibiting diverse shapes and sizes (Blaschke et al., 2004). SegFormer’s multi-scale feature fusion module can simultaneously focus on fine-grained local information and global contextual relationships, significantly improving segmentation performance for small objects and boundaries (Chen et al., 2024). Therefore, the selection of SegFormer primarily considers its ability to demonstrate higher segmentation accuracy and robustness when processing remote sensing images with complex backgrounds, multi-scale targets, and diverse land cover types. It is particularly suitable for semantic segmentation tasks involving large-scale, high-resolution remote sensing images, such as urban building areas, farmland distribution, and mixed forest and water bodies. Its structural diagram is shown in **Figure 3**. C is the number of channels, and Decoder fuses these features through MLP.

### 3.2 Improved SegFormer remote sensing image nectar source segmentation model

**Figure 4:**
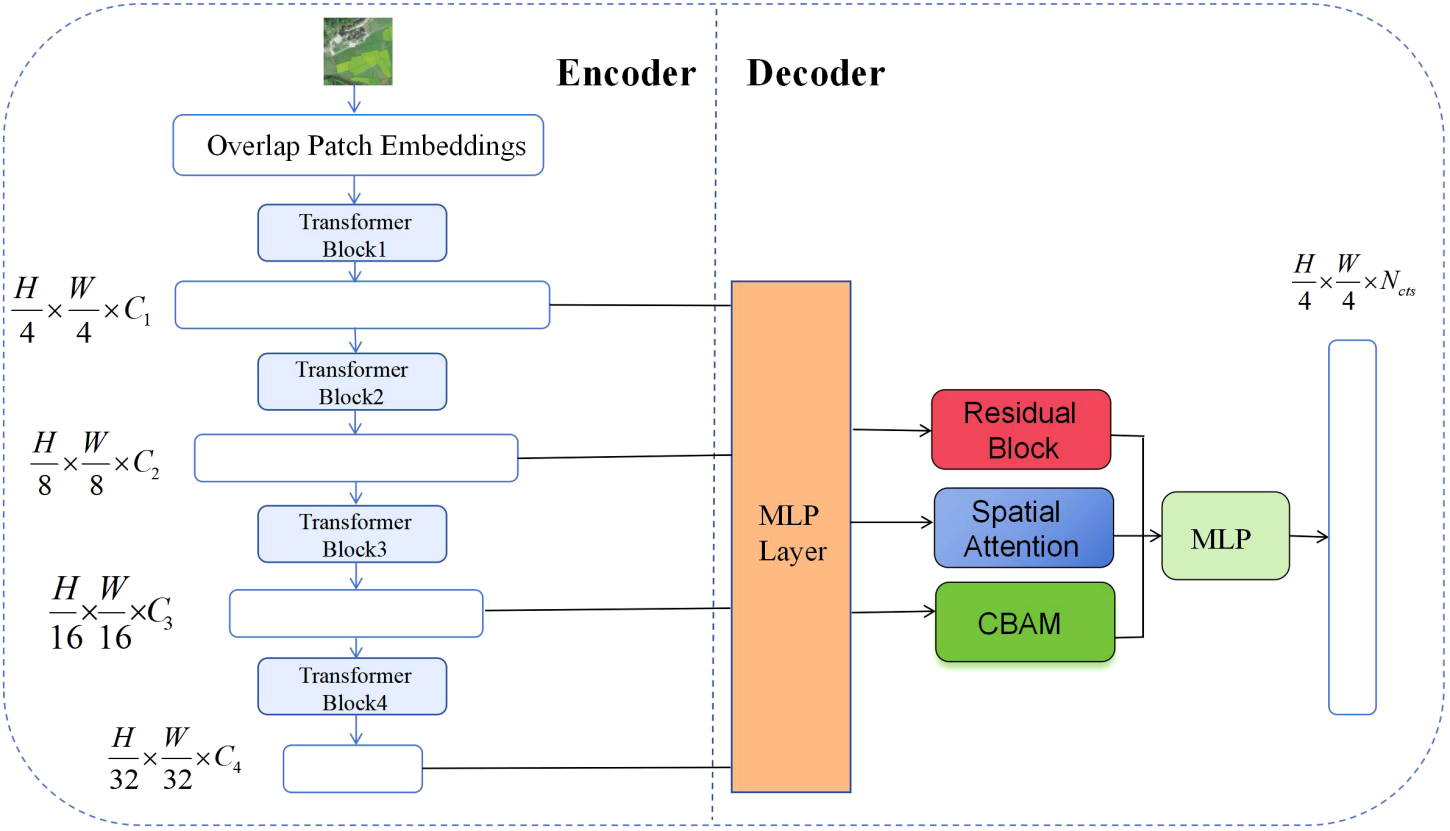
Structural diagram of the improved SegFormer remote sensing image nectar source segmentation model.

To improve the performance of the SegFormer model in the task of segmenting nectar-producing plants in remote sensing images, this paper proposes targeted structural improvements to the model. The overall improvement framework is shown in **Figure 4**. While retaining the original encoder-decoder backbone structure, this paper sequentially introduces the CBAM attention mechanism, deep residual structure, and spatial attention module during the feature fusion stage. This approach not only effectively extracts key information from multi-scale features but also significantly enhances the model’s segmentation capabilities for multifaceted scenes and fine-grained targets.

#### 3.2.1 CBAM attention

**Figure 5:**
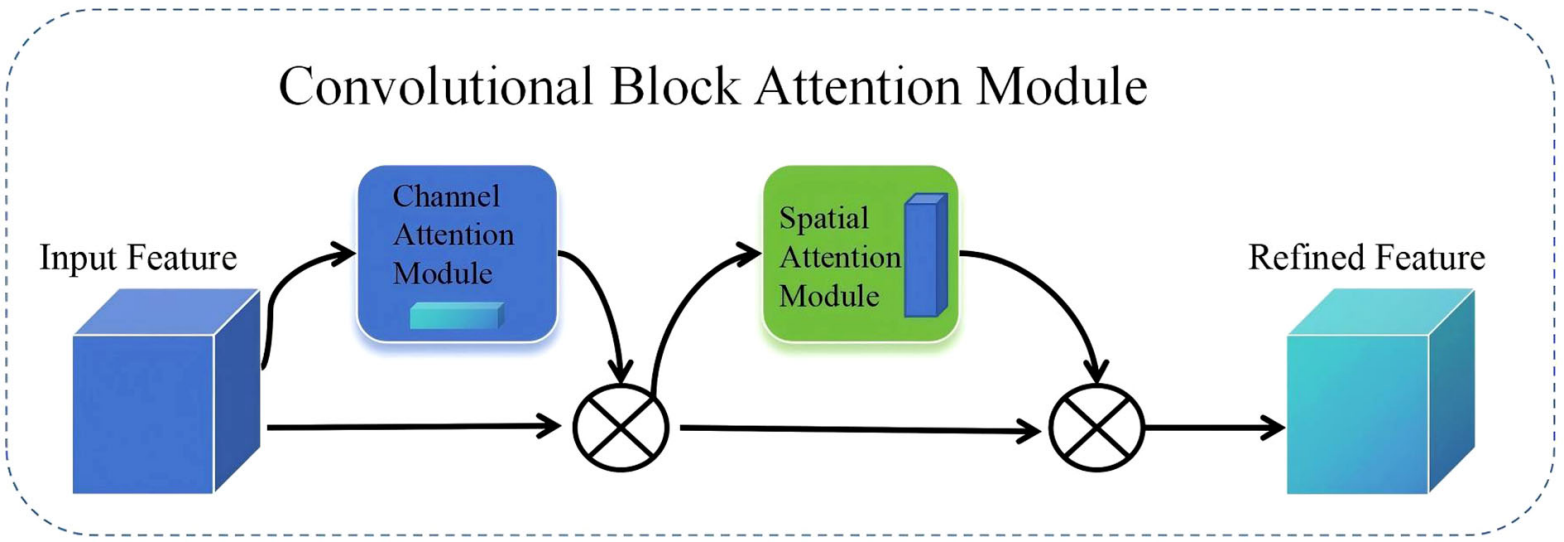
CBAM structure diagram.

To further enhance the model’s feature extraction capabilities, this paper introduces the CBAM (Convolutional Block Attention Module) attention mechanism into the feature fusion module of SegFormer. CBAM is a lightweight, pluggable attention mechanism proposed by Sanghyun Woo et al. in their 2018 ECCV paper titled “CBAM: Convolutional Block Attention Module.” (Woo et al., 2018) CBAM can adaptively adjust feature responses in both the channel and spatial dimensions, thereby enhancing the network’s ability to express key information. In this study, we integrate the CBAM module into the SegFormer framework to enhance the model’s feature extraction and fine-grained object discrimination capabilities in the task of honey plant segmentation in remote sensing images. CBAM primarily consists of a channel attention module and a spatial attention module, which are used in series (Wang et al., 2021), as shown in **Figure 5**.

Channel attention applies global average and max pooling to input features, generating two vectors, which are summed and passed through a Sigmoid function to obtain attention weights. These weights adjust the contribution of each channel, emphasizing important information. The spatial attention module pools along the spatial dimension, concatenates results, and applies a convolutional layer followed by a Sigmoid function to get spatial attention weights. Equations 1 and 2. Compared to SENet, BAM, and ECA, enhances both spatial and channel focus, making it especially effective for complex remote sensing images in nectar plant segmentation tasks. The formulas are given in Equation.


(1) $$M_{c}\left(F\right)=\sigma \left(\text{MLP}\left(\text{AvgPool}\left(F\right)\right)+\text{MLP}\left(\text{MaxPool}\left(F\right)\right)\right)$$

(2) $$M_{s}\left(F\right)=\sigma \left(f^{7\times 7}\left(\left[\text{AvgPool}\left(F\right);\;\text{MaxPool}\left(F\right)\right]\right)\right)$$

*M_c_* (*F*) is the channel attention weight. An adaptive coefficient between 0 and 1 is assigned to each channel. *F* represents the input features or image data. AvgPool(*F*) is global average pooling, MaxPool(*F*) is global maximum pooling, and *σ* refers to the Sigmoid activation function.

#### 3.2.2 Depth residual

**Figure 6:**
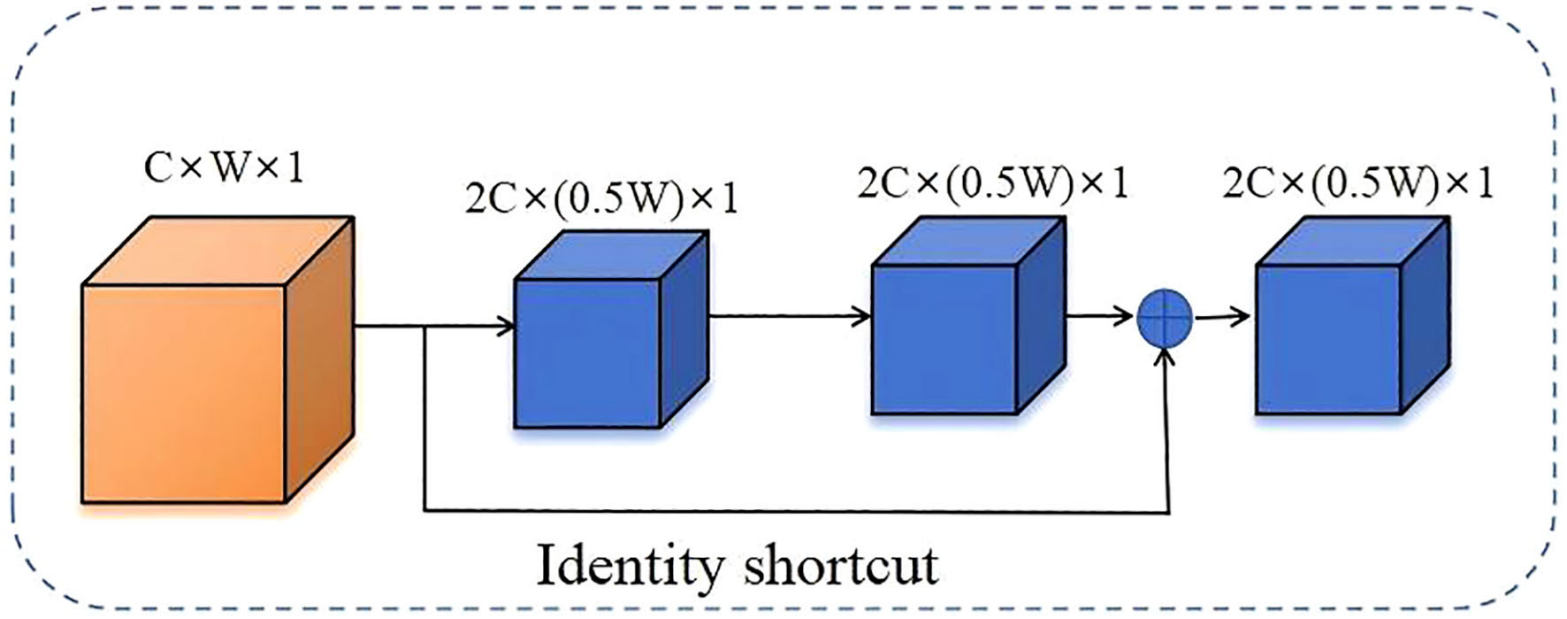
Diagram of the deep residual structure with identity shortcut and convolutional layers.

To enhance the model’s adaptability and feature representation capabilities in complex scenarios, this paper introduces a deep residual structure into the feature fusion stage of SegFormer (as shown in **Figure 6**). The deep residual structure was first proposed by He et al. and has since become a core module in modern deep neural networks (He et al., 2016). Its fundamental idea is to add a shortcut path with an identity mapping alongside the main branch, enabling direct feature transmission and superposition, thereby improving the efficiency of information flow within the network. Compared to traditional structures, residual structures not only effectively preserve input features, but also significantly enhance the network’s ability to model complex targets and fine-grained features, providing a robust foundation for improving segmentation accuracy and robustness (Lin et al., 2017).

The deep residual structure uses shortcuts through identity mappings to directly add input features to output features that have undergone several convolutions, normalizations, and activations (He et al., 2016; Yu et al., 2018). This not only deepens the number of network layers but also effectively alleviates the problems of gradient disappearance and network degradation. The mathematical expression is as follows Equation 3.


(3) $$Y=M_{s}\left(F\right)⊗F$$

*x* is the input feature, *F*(*x*) represents the residual mapping to be learned, and *y* represents the desired original mapping.

#### 3.2.3 Spatial attention

In this study, although the CBAM module has integrated channel attention and spatial attention mechanisms, enabling adaptive enhancement of feature representation in different dimensions, the complex background information and multi-dimensional distribution of fine-grained targets in remote sensing images still pose substantial challenges for spatial feature modeling. To further enhance the model’s sensitivity to key spatial regions, this paper introduces an independent spatial attention mechanism based on the CBAM module.

**Figure 7:**
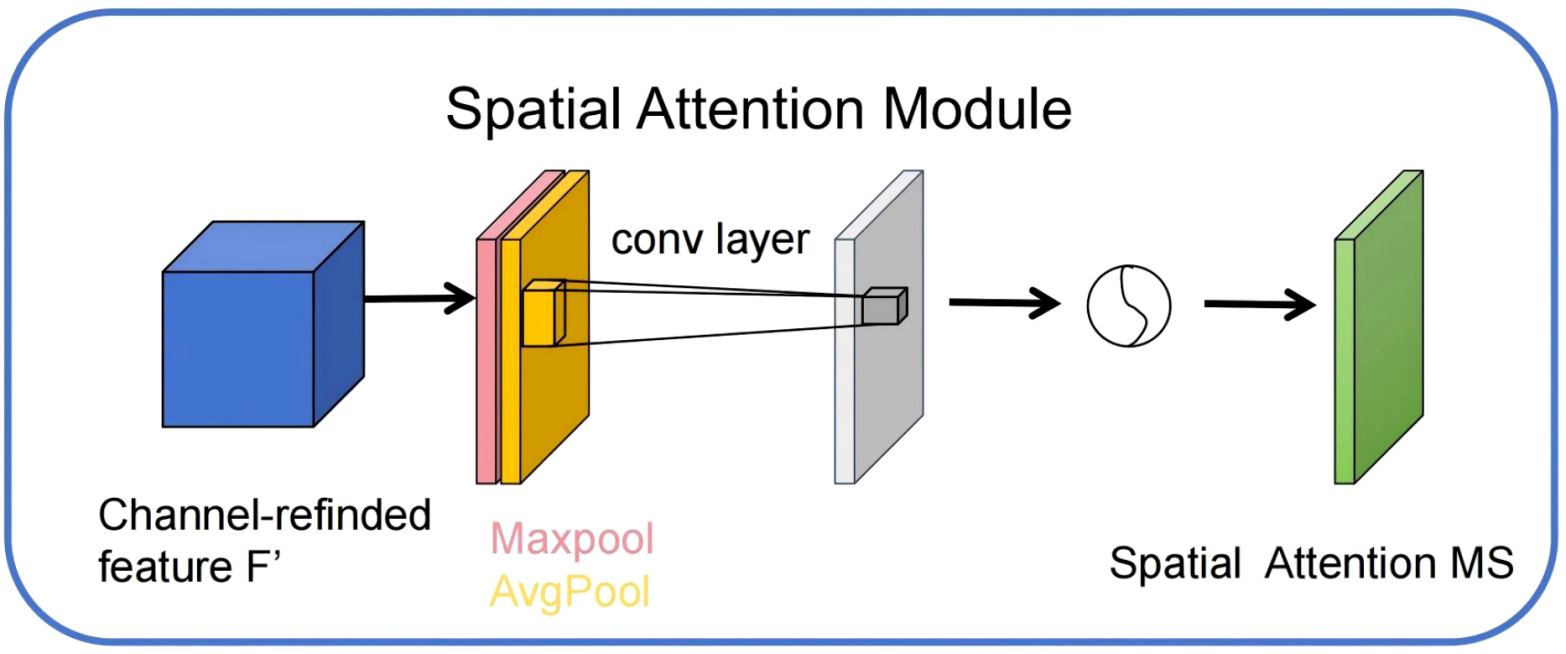
Independent spatial attention structure diagram.

The spatial attention mechanism primarily targets the spatial position dimension of feature maps, guiding the model to focus on key regions related to the segmentation target by adaptively learning response weights for different positions (Gu et al., 2020). Specifically, spatial attention first performs global average pooling and global max pooling on the input features in the channel dimension, yielding two two-dimensional maps representing spatial distribution features. These two maps are then concatenated along the channel dimension, followed by feature fusion through a convolutional layer (Chen et al., 2017). A Sigmoid activation function is used to generate the spatial attention weight map. This weight map applies element-wise weighting to the original features, significantly enhancing important spatial regions while effectively suppressing irrelevant regions. The structural diagram is shown in **Figure 7** below.

## 4 Results

### 4.1 Experiment running platform

This study was conducted on the Windows 11 operating system, equipped with a 12th-generation Intel Core i7 processor, an NVIDIA GeForce RTX 3090 graphics card, and using Python 3.8 with PyTorch 1.11.0 and CUDA version 11.3. The study utilized software packages including NumPy, Pandas, Matplotlib, Seaborn, OpenCV, and torchvision, along with the SegFormer model to perform remote sensing image segmentation tasks. CUDA provides GPU acceleration, ensuring efficient model training and evaluation.

### 4.2 Evaluation indicators

To comprehensively evaluate the performance of the improved SegFormer model in the task of segmenting nectar-producing plants in remote sensing images, this article selected four evaluation metrics: mean intersection over union (mIoU), mean pixel accuracy (mPA), mean precision (mPrecision) and mean recall (mRecall). Their mathematical expressions are as follows Equations 4–7.


(4) $$\text{mIoU}=\frac{1}{K}\sum_{k=1}^{K}\frac{TP_{k}}{TP_{k}+FP_{k}+FN_{k}}$$

(5) $$\text{mPA}=\frac{1}{K}\sum_{k=1}^{K}\frac{TP_{k}}{TP_{k}+FN_{k}}$$

(6) $$\text{mPrecision}=\frac{1}{K}\sum_{k=1}^{K}\frac{TP_{k}}{TP_{k}+FP_{k}}$$

(7) $$\text{mRecall}=\frac{1}{K}\sum_{k=1}^{K}\frac{TP_{k}}{TP_{k}+FN_{k}}$$

Where *K* denotes the total number of categories, *TP_k_* denotes the true positives of category *k*, *FP_k_* denotes the false positives of category *k*, and *FN_k_* denotes the false negatives of category *k*.

### 4.3 Ablation experiments

To validate the role of each module in the improved SegFormer model for segmentation tasks on remote sensing images, this paper designed multiple ablation experiments and compared and evaluated each model under the condition of 100 training iterations. The specific results are shown in **Table 1**. As shown in the table, the baseline model (ID 0) without any modules achieved an mIoU of 89.31%, an mPA of 94.15%, and both mPrecision and mRecall at 94.15%. By introducing the CBAM attention mechanism (ID 1), the deep residual module (ID 2), and the spatial feature module (ID 3), the model performance improved. Specifically, after adding CBAM, mIoU improved to 90.43% and mPA to 94.64%; after introducing the deep residual module, mIoU and mPA reached 90.80% and 95.00%, respectively; while introducing only the spatial module resulted in an mIoU of 89.88%, with limited improvement.

**Table 1:** Ablation experiment comparison table.

Serial number	CBAM attention	Depth residual	Spatial attention	mIoU/%	mPA/%	mPrecision/%	mRecall/%
0				89.31	94.15	94.25	94.15
1	✓			90.43	94.64	95.07	94.64
2		✓		90.80	95.00	95.14	95.00
3			✓	89.88	94.62	94.43	94.62
4	✓	✓		90.76	94.90	95.18	94.90
5	✓		✓	90.53	94.85	94.97	94.85
6		✓	✓	90.68	94.75	95.24	94.75
7	✓	✓	✓	91.05	95.02	95.40	95.02

“✓” indicates that this module has been added.

**Figure 8:**
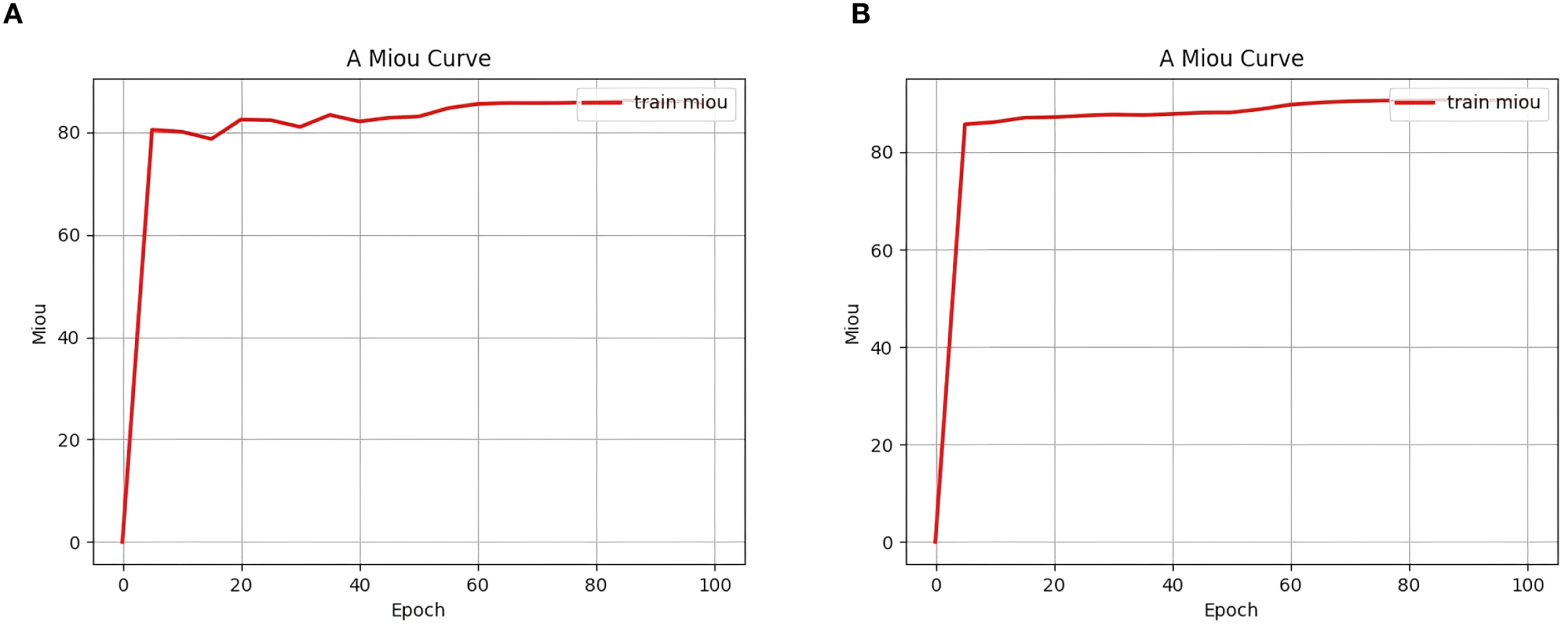
Comparison of initial mIoU change curves. The y-axis of this figure represents the mIoU, the x-axis represents the number of training epochs, and the mIoU curve for the training set is marked in red. **(a)** represents the “Item 0 experiment” and **(b)** represents the ”Item 7 experiment”.

Next, by combining the various modules, it can be observed that when CBAM and the deep residual module (item 4) are added together, the mIoU improves to 90.76% and the mPA improves to 94.90%; When CBAM and the spatial module (item 5) are combined, mIoU is 90.68%; when both the deep residual and spatial modules (item 6) are added, mIoU is 90.68%, and mPA is 94.75%. Finally, when all three modules are combined (item 7), the model achieves optimal performance after 100 training rounds, with mIoU reaching 91.05%, mPA improving to 95.02%, and mPrecision and mRecall reaching 95.40% and 95.02%, respectively. Additionally, as shown in **Figure 8**, the seventh model converged faster in terms of mIoU during training and achieved a higher final value than the baseline model, further validating the improvement of segmentation performance by the enhanced modules. The combined use of CBAM, deep residuals, and spatial modules can greatly improve the segmentation performance of the model. Each module has a positive effect on the model, and the combined effect of all modules achieves optimal results.

### 4.4 Comparative experiments

To comprehensively evaluate the segmentation performance of the proposed improved SegFormer model, this paper conducted comparative experiments with various mainstream segmentation methods, including Unet, Pspnet, Hrnet, Deeplabv3, and the original SegFormer. The results are shown in **Table 2**. As can be seen, the improved SegFormer achieves the best performance across all metrics, significantly outperforming other comparison models, indicating that the proposed method demonstrates superior accuracy and generalization capabilities in remote sensing image segmentation tasks. Additionally, the original SegFormer also outperforms traditional convolutional neural network models, further highlighting the advantages of the Transformer architecture in semantic segmentation tasks.

**Table 2:** Comparison of test results between SegFormer before and after improvement and other models.

Model	mIoU/%	mPA/%	mPrecision/%	mRecall/%
Unet	87.68	92.73	93.72	92.73
TransUnet	90.44	95.19	94.53	95.19
SwinUnet	87.39	93.88	93.46	93.88
Pspnet	85.89	91.40	92.87	91.40
Hrnet	87.95	93.74	93.05	93.74
Deeplabv3	86.44	92.18	92.75	92.18
SegFormer	89.31	94.15	94.25	94.15
Improved SegFormer	91.05	95.02	95.40	95.02

**Figure 9:**
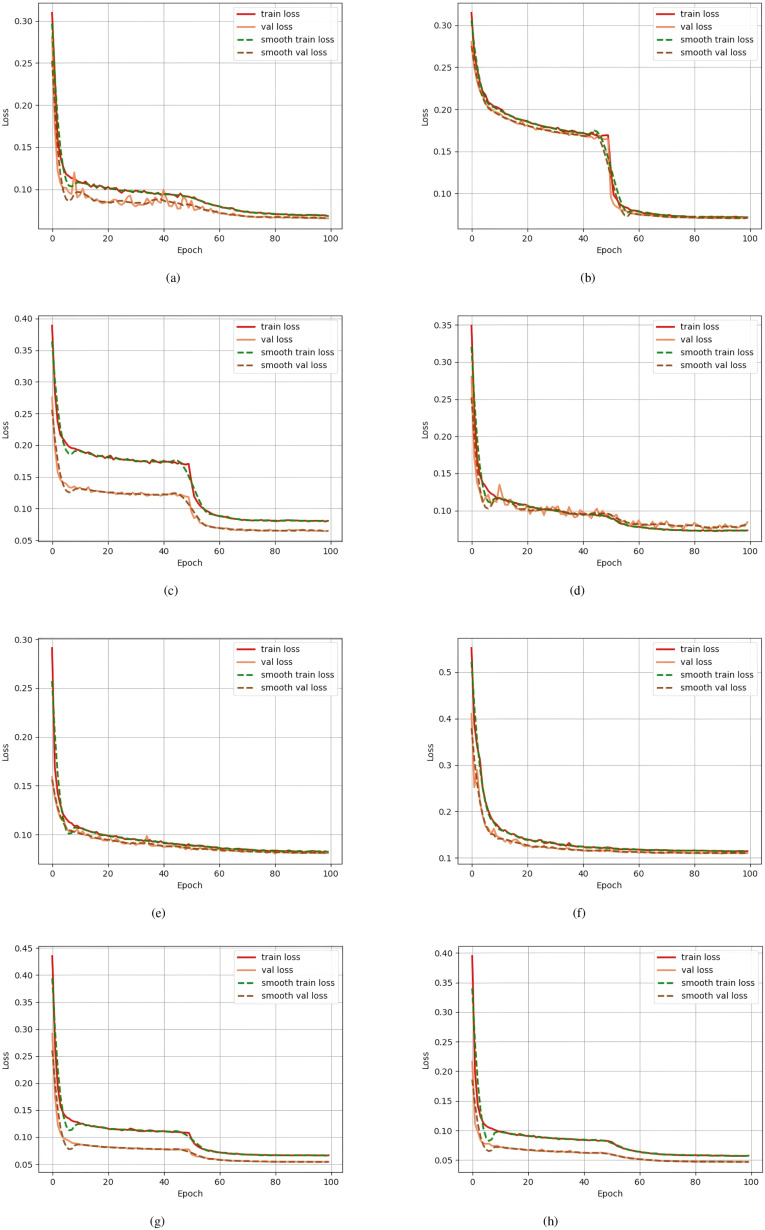
Comparison of loss change curves during training for each model. The y-axis represents the Loss, the x-axis represents the number of training epochs, and different loss curves (train loss, val loss, smooth train loss, smooth val loss) are represented by different colors. **(a)** corresponds to “Unet”, **(b)** to “Pspnet”, **(c)** to “Hrnet”, **(d)** to “Deeplabv3”, and **(e)** to “TransUnet”; the previous label for **(f)** contained an error, and it has now been corrected to **(f)** corresponding to “SwinUnet”; in addition, **(g)** corresponds to “SegFormer” and **(h)** to “Improved SegFormer”.

In addition, **Figure 9** compares the loss change curves of each model during training. It can be seen that the improved SegFormer converges to a lower level faster in both the training and validation sets, and the training loss and validation loss always maintain a small gap. The above experimental results fully demonstrate that the proposed improved SegFormer can not only effectively improve the accuracy of remote sensing image segmentation, but also has better convergence and generalization capabilities.

**Figure 10:**
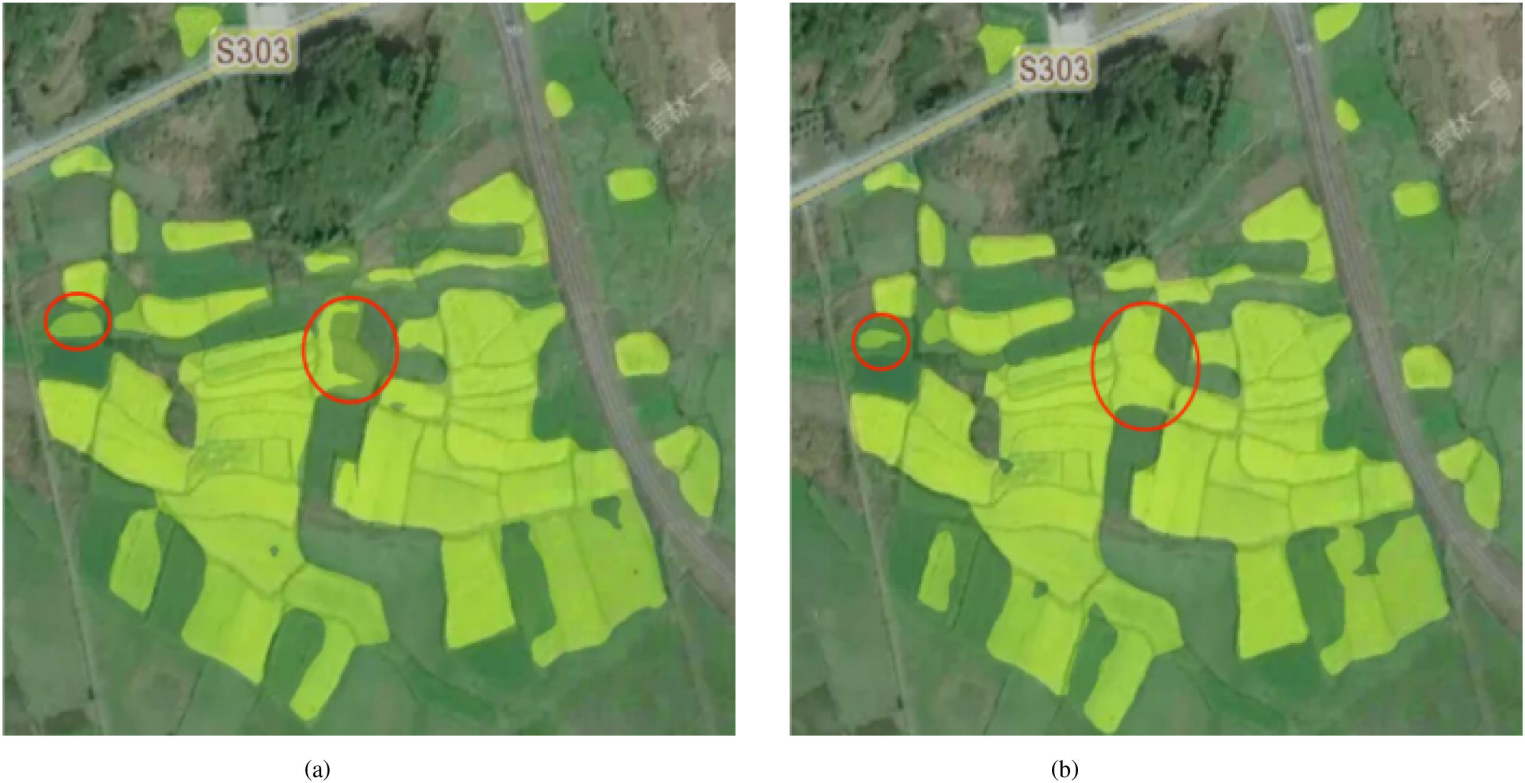
Comparison of prediction results before and after improvement. This is a remote sensing image, showing the changes in the target area and the effect after model processing, with the red markings indicating areas of significant change. **(a)** shows the prediction result of “SegFormer” and **(b)** shows the prediction result of the “Improved SegFormer”.

**Figure 10** shows a comparison of the segmentation results of the original SegFormer model and the improved SegFormer model on the same remote sensing image. It can be seen that the improved model achieves more accurate and complete segmentation of field boundaries and small-area target regions, with a significant reduction in missed and misclassified areas marked by red circles. This result not only improves the accuracy of automatic identification of nectar-producing plants but also provides strong support for the practical application of remote sensing technology in fields such as nectar resource surveys and ecological environment monitoring.

## 5 Discussion

To address the segmentation requirements of remote sensing images for Wuyuan County’s complex terrain and diverse distribution of nectar-producing plants, this paper proposes an improved SegFormer segmentation method using rapeseed flowers as a representative nectar-producing plant. The method integrates the CBAM attention mechanism, deep residual structure, and spatial attention module. Through experiments on multi-source remote sensing image datasets, the model demonstrates superior performance compared to mainstream segmentation methods such as UNet, Pspnet, Hrnet, Deeplabv3, and the original SegFormer in nectar plant boundary identification, segmentation of scattered and small-scale targets, and other aspects. It enables more precise and reliable extraction of spatial distribution features.

Experimental results show that the improved model achieves significant improvements in key evaluation metrics such as mIoU and mPA, better addressing complex terrain backgrounds and seasonal changes in nectar-producing plants. The model performs particularly well in areas such as the edges of rapeseed flower fields and sparsely distributed regions. Based on this method, not only can the efficient and accurate identification of nectar-producing plants in remote sensing images be realized, but it also provides beekeepers with scientific information on nectar distribution, assisting in beekeeping decisionmaking and enhancing honey production and quality. Additionally, the method offers productive and intelligent technical support for practical applications such as nectar resource surveys, precision beekeeping management, and dynamic monitoring of ecological environments, effectively overcoming the limitations of traditional manual surveys, which are time-consuming, labor-intensive, and lack timeliness.

However, this study has limitations. First, while the improved model performed well on rapeseed flower images from Wuyuan County, its generalizability remains insufficiently verified for other nectarproducing plants or images from diverse regions. Second, the model’s real-time deployment performance may be affected by factors like remote sensing image resolution, data processing speed, and hardware conditions, particularly in large-scale and dynamic settings. Third, although the method enhances nectarproducing plant segmentation efficiency, it depends on high-resolution remote sensing imagery, potentially constrained by data acquisition and processing costs in some scenarios. To address these limitations, future work will focus on several key directions: testing the method on other nectar-producing plant species (e.g., lavender, sunflower) and under different environmental conditions (such as arid, tropical regions) to assess generalizability; conducting deployment and field tests in actual agricultural and ecological monitoring scenarios through collaboration with local agencies and enterprises, evaluating performance in real-world dynamic conditions; adapting the model to different growth stages (seedling, flowering, fruiting) and stress conditions (drought, pest infestation) by incorporating stage-specific and stress-related features into training; and thoroughly assessing scalability by comparing performance on smaller plots versus larger commercial farms, a critical aspect for practical application given its direct impact on utility in large-scale scenarios. These efforts will enhance the model’s practicality and application scope, making it more robust and versatile for nectar-producing plant segmentation tasks.

## Data Availability

The raw data supporting the conclusions of this article will be made available by the authors, without undue reservation.
