# Supplementary figures and images for: SegFormer-based nectar source segmentation in remote sensing imagery
Source: Front Plant Sci. 2025 Oct 1;16:1666619. doi: 10.3389/fpls.2025.1666619 (PMC12521987; doi:10.3389/fpls.2025.1666619)

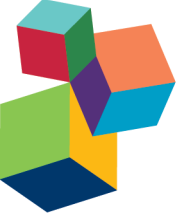

frontiers

Supplement: Supplementary file 1 [file DataSheet1.zip › logo1.pdf]

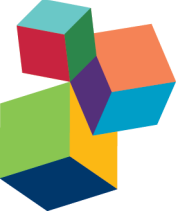

Supplement: Supplementary file 1 [file DataSheet1.zip › logo2.pdf]

A

frontiers  
FOR YOUNG MINDS

B

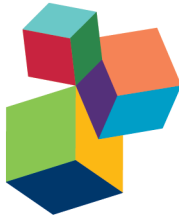

Supplement: Supplementary file 1 [file DataSheet1.zip › logos.pdf]

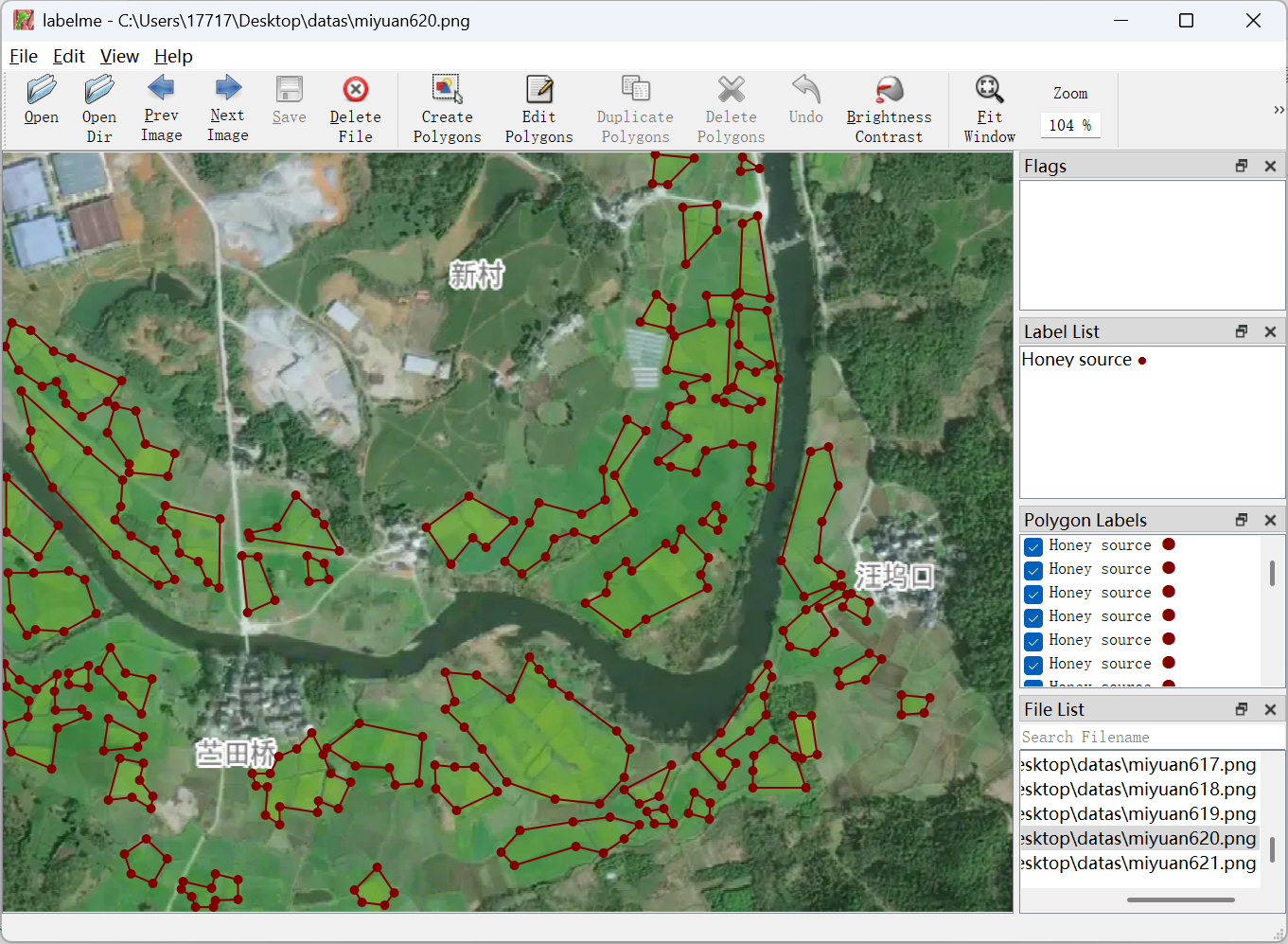

Supplement: Supplementary file 1 [file DataSheet1.zip › FIG2.jpg]

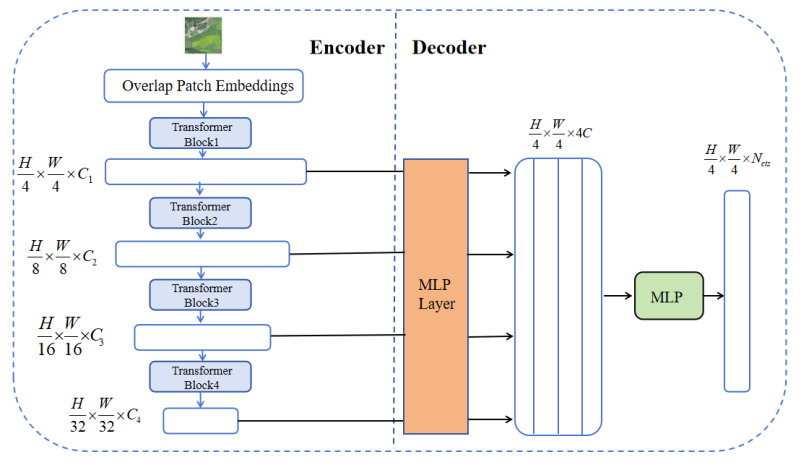

Supplement: Supplementary file 1 [file DataSheet1.zip › FIG3.jpg]

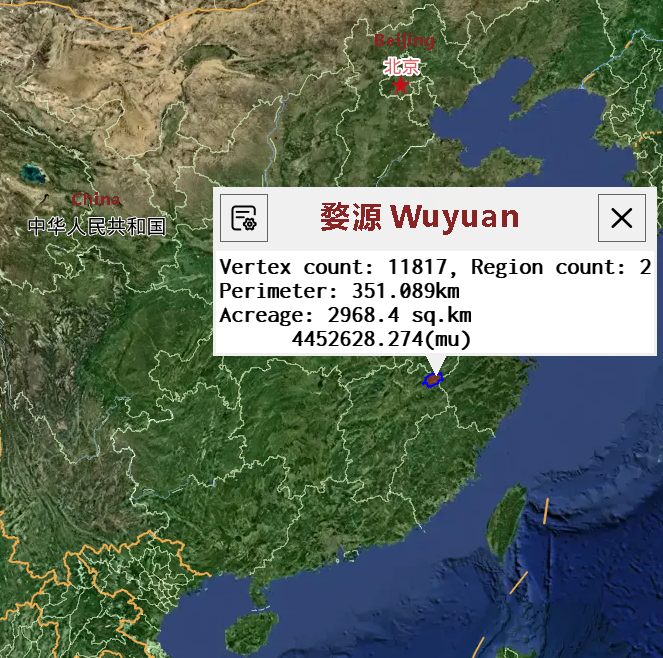

Supplement: Supplementary file 1 [file DataSheet1.zip › FIG1.png]

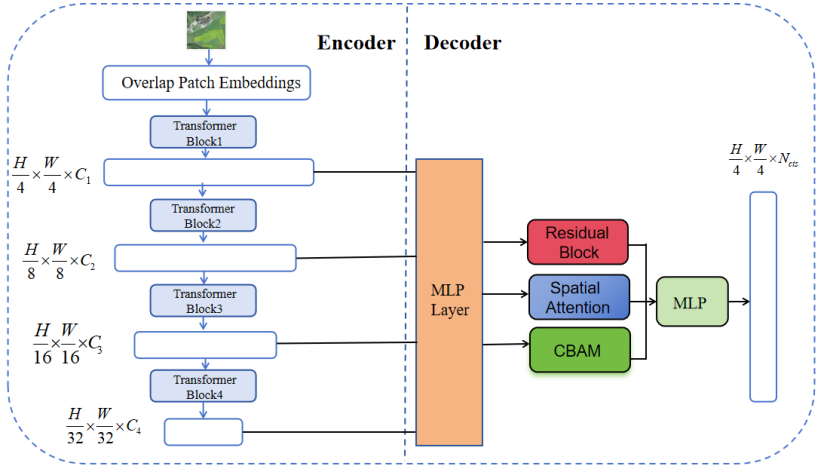

Supplement: Supplementary file 1 [file DataSheet1.zip › FIG4.jpg]

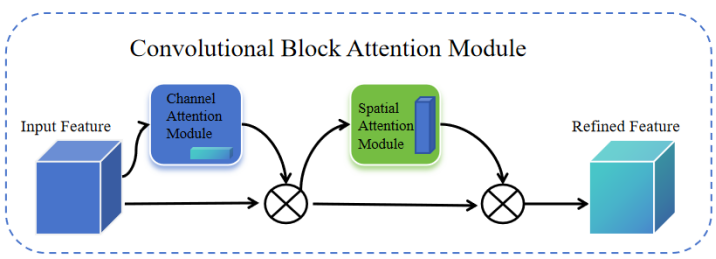

Supplement: Supplementary file 1 [file DataSheet1.zip › FIG5.jpg]

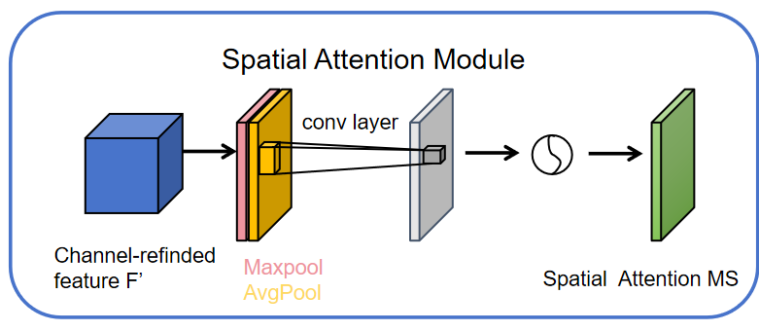

Supplement: Supplementary file 1 [file DataSheet1.zip › FIG7.jpg]

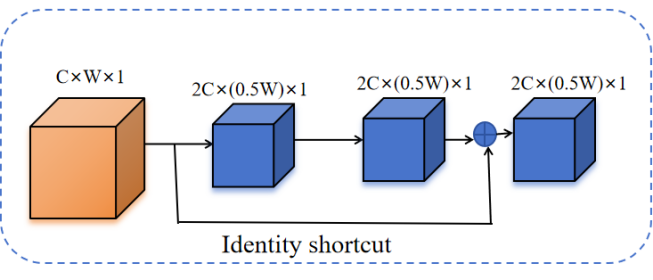

Supplement: Supplementary file 1 [file DataSheet1.zip › FIG6.jpg]

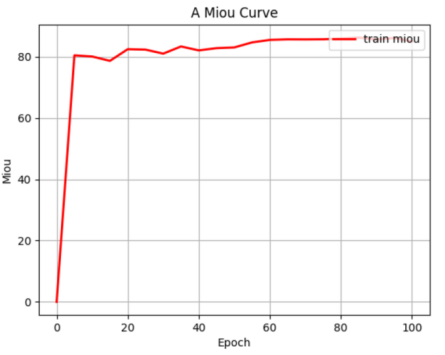

Supplement: Supplementary file 1 [file DataSheet1.zip › FIG8-1.jpg]

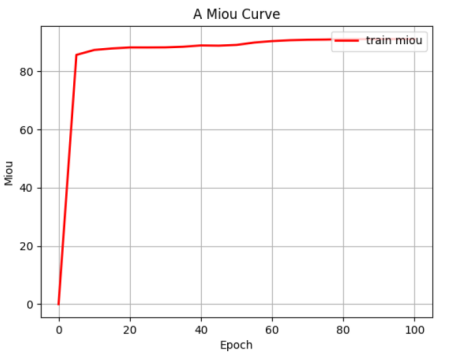

Supplement: Supplementary file 1 [file DataSheet1.zip › FIG8-2.jpg]

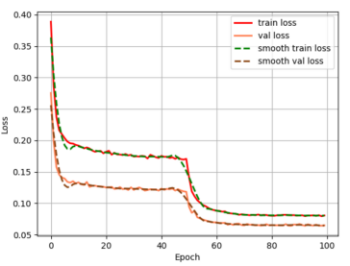

Supplement: Supplementary file 1 [file DataSheet1.zip › FIG9-2.jpg]

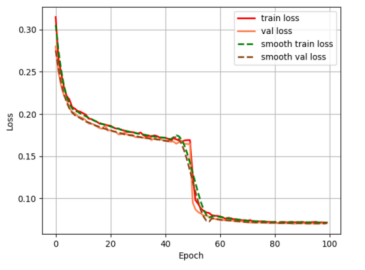

Supplement: Supplementary file 1 [file DataSheet1.zip › FIG9-3.jpg]

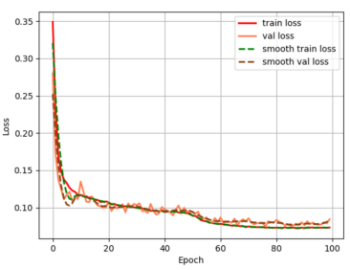

Supplement: Supplementary file 1 [file DataSheet1.zip › FIG9-1.jpg]

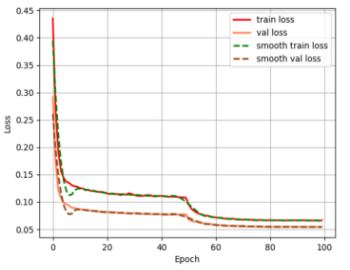

Supplement: Supplementary file 1 [file DataSheet1.zip › FIG9-4.jpg]

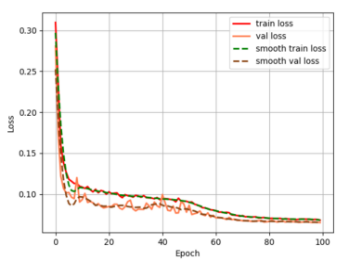

Supplement: Supplementary file 1 [file DataSheet1.zip › FIG9-5.jpg]

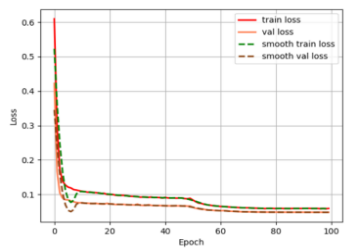

Supplement: Supplementary file 1 [file DataSheet1.zip › FIG9-6.jpg]

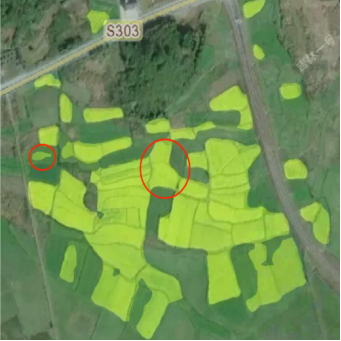

Supplement: Supplementary file 1 [file DataSheet1.zip › FIG10-2.jpg]

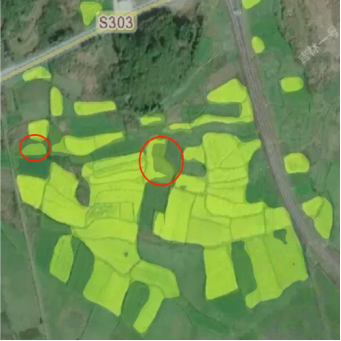

Supplement: Supplementary file 1 [file DataSheet1.zip › FIG10-1.jpg]
